# “Small Thyroid Gland” in Reproductive Women without Autoimmune Thyroid Disease—Ultrasonographic Evaluation as a Useful Screening Tool for Hypothyroidism

**DOI:** 10.3390/jcm10091828

**Published:** 2021-04-22

**Authors:** Justyna Milczarek-Banach, Piotr Miśkiewicz

**Affiliations:** Department of Internal Medicine and Endocrinology, Medical University of Warsaw, Banacha 1a, 02-097 Warsaw, Poland; justyna.banach@uckwum.pl

**Keywords:** thyroid volume, hypothyroidism, thyrotropin, reproduction, women, small thyroid gland

## Abstract

Proper thyroid function is important for women of childbearing age, as hypothyroidism affects fertility, pregnancy and offspring. The upper reference limit for thyrotropin (TSH) in pregnancy was defined as <2.5 mU/L in the first trimester. Recommendations include either universal screening of TSH before pregnancy, or identifying individuals at “high risk” for thyroid illness. “Small thyroid gland” not associated with autoimmune thyroid disease (AITD) seems to be a reason for hypothyroidism and probably should be included in target case finding procedure before pregnancy. The purpose of this cross-sectional study was to analyze relationships between the thyroid volume and its function, and to determine the thyroid volume as a predictive factor for TSH levels above 2.5 µIU/mL in reproductive women without AITD. We included 151 women without AITD, and aged 18–40. Blood and urine samples were analyzed for parameters of thyroid function. Ultrasound examination of the thyroid was performed. The thyroid volume was negatively correlated with TSH. Women with a thyroid volume in the 1st quartile for the study population presented higher TSH levels versus women in the 4th quartile (*p* = 0.0132). A thyroid volume cut-off point of 9 mL was the predictive factor for TSH levels above 2.5 µIU/mL (*p* = 0.0037).

## 1. Introduction

Proper functioning of a thyroid gland is essential for maintenance of hormonal homeostasis in human beings. Euthyroidism is especially important for women of reproductive age, as hypothyroidism affects fertility, pregnancy outcomes, and the health of offspring. Subclinical and overt hypothyroidism in pregnancy may lead to preeclampsia, premature delivery, premature abruption of the placenta and miscarriage [1,2,3]. Maternal hypothyroidism can cause disturbances of intrauterine growth of the offspring, low birth weight and the retardation of neural development of the fetus [1,2,3]. According to the American Thyroid Association guidelines for the diagnosis and management of thyroid disease during pregnancy and postpartum from 2011; and the European Thyroid Association guidelines for the management of subclinical hypothyroidism in pregnancy and in children from 2014; the upper reference limit for thyrotropin (TSH) in pregnancy was defined as <2.5 mU/L in the first trimester, and <3.0–3.5 mU/L in the second and third trimesters [4,5]. Recommendations given by different endocrine societies include either universal screening of TSH before pregnancy, or identifying individuals at “high risk” for thyroid illness [4,5,6]. “Small thyroid gland” not associated with autoimmune thyroid disease (AITD), seems to be the reason for hypothyroidism in some patients [7]. It is worth considering including women with “small thyroid gland” in target case finding procedure before pregnancy.

Several studies have reported that the thyroid volume depends on: age, weight, height, body mass index (BMI) or body surface area (BSA) [8,9,10]. Of course, the thyroid volume could not be entirely explained by anthropometric measurements. It is also determined by other factors, such as ethnicity, genetic background and environmental factors (smoking, nutrition, iodine intake) [10,11,12,13]. These could be the reasons for the variations of the results in different geographic areas [8,9,10,14]. Therefore, the authors of the previous reports suggested the need for population-specific references.

The aim of this study was to analyze the potential relationships between the thyroid volume and the laboratory parameters of thyroid function. Moreover, the purpose of this study was to determine the lower reference limit for the thyroid volume in women of childbearing age without AITD, living in Warsaw, Poland.

## 2. Materials and Methods

### 2.1. Patients

This cross–sectional study was performed in the Department of Internal Medicine and Endocrinology, Medical University of Warsaw, Warsaw, Poland, in the period between October 2017 and May2019. After providing written informed consent to participate, we included women aged 18–40 years in the study. Women were excluded from the study if they fulfilled at least one of the following criteria: (i) documented history of any thyroid disease (hypothyroidism, hyperthyroidism, thyroid nodules, autoimmune thyroid disease); (ii) pharmacotherapy influencing the thyroid function (especially medicines consisting of: iodine, antithyroid drugs, levothyroxine, selenium); (iii) positive thyroid peroxidase antibodies (TPOAb) and/or thyroglobulin antibodies (TGAb) during this study; (iv) hypoechoic—inhomogenous thyroid gland in ultrasound; (v) pregnancy or puerperium. In total, 151 women were recruited into the study. Basal characteristics of the participants are presented in Table 1.

### 2.2. Laboratory Measurements

Venous blood was drawn from all the study participants, in the morning, after overnight fasting (>8 h). After 30 min of incubation at room temperature, the blood samples were centrifuged for 15 min (2500 rpm). Then, serum samples were frozen and stored vertically at −70 °C for further analyses.

TSH, free thyroxine (fT4), TPOAb and TGAb were measured in serum samples using an electrochemiluminescence immunoassay (Roche Diagnostics, Mannheim, Germany) on a Cobas e411 Analyzer (Hitachi, Tokyo, Japan), in the Scientific Laboratory at the Department of Internal Medicine and Endocrinology, Medical University of Warsaw. Serum TPOAb and TGAb >34 IU/mL and >115 IU/mL respectively, were considered as “positive”.

Moreover, each study participant brought a 100 mL sample of midstream morning urine for the measurement of urinary iodine and creatinine. Urine iodine concentrations were estimated by the catalytic arsenium-cerium method based on Sandell–Kolthoff reaction [15]. Urinary creatinine was measured by colorimetric Jaffe method [16].

### 2.3. Ultrasound of the Thyroid

Ultrasound of the thyroid gland was conducted using a Hitachi Avius Medical ultrasound technique system equipped with the 3–15 MHz high frequency linear array transducer. Thyroid ultrasound procedures were done on the basis of the Ultrasound Examination Standards of the Polish Ultrasound Society and American College of Radiology Guidelines [17,18]. Participants were lying in a supine position with the neck hyperextended. Thyroid volume was calculated with a simplified formula for the volume of a spheroid [17]: V = 0.5 × W × H × L, where V—volume of the lobe, 0.5—simplified coefficient, W—width, H—height and L—length. The total thyroid volume was obtained by combining the volumes of left and right lobes. The isthmus was not taken into account. All ultrasonographic examinations were performed and interpreted by the same physician with 5-years of experience (JMB).

### 2.4. Study Design

The analysis of these data was performed in the following steps:

- Analysis of the relationship between the thyroid volume and the thyroid function [TSH, fT4, ioduria, ioduria/creatinine (I/CR)].
- Determination of the lower reference limit for the thyroid volume, for estimating if the level of TSH is >2.5 µIU/mL.

### 2.5. Statistical Analysis

Distributions of the variables were estimated using the Shapiro–Wilk test. Continuous variables were demonstrated as arithmetic means ± standard deviation (SD). Categorical data were shown as numbers and percentage values (%). Correlations between the continuous data (thyroid volume, TSH, fT4, ioduria, I/CR) were calculated with the Spearman correlation coefficient or Pearson correlation test. Additionally, the multivariate linear regression was performed. Comparisons of continuous data (thyroid volume, TSH, fT4, ioduria, I/CR) in two independent groups of participants (with thyroid volume in the 1st quartile (Q1) vs. 4th quartile (Q4) for the study population) were performed with the Mann–Whitney U test or Student t test respectively, for non-normal and normal distribution of these data. Results with a *p* value of <0.05 were considered to present statistical significance. The determination of the lower reference limit for the thyroid volume was done with the receiver operating characteristic (ROC) curve with the estimation of sensitivity, specificity and accuracy of the thyroid volume thresholds for the limit of TSH > 2.5 µIU/mL.

## 3. Results

### 3.1. Relationship between the Thyroid Volume and the Thyroid Function

**Figure 1:**
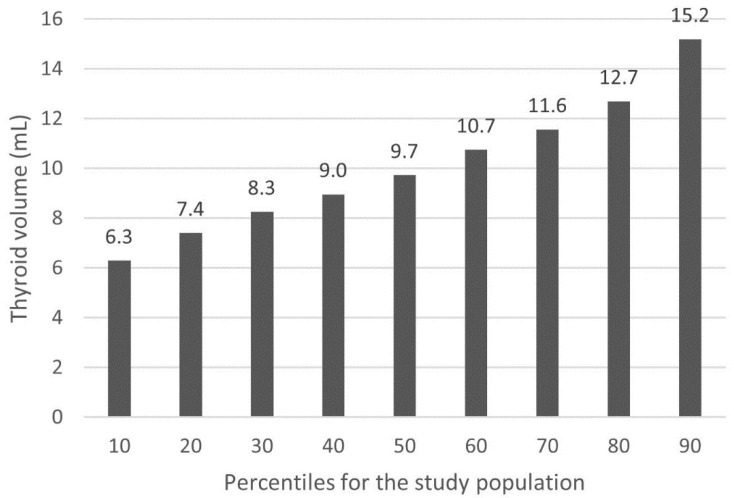
Percentiles of the thyroid volume for the study population.

The quartiles of the thyroid volume for the study population were as follows: 1st quartile (Q1): 4.0–7.9 mL; 2nd quartile (Q2): 8.0–9.7 mL; 3rd quartile (Q3): 9.8–12.2 mL; 4th quartile (Q4): 12.3–20.4 mL. All the results of the calculations of percentiles for the thyroid volume in the study population are shown in Figure 1.

All correlations between the thyroid volume and the laboratory parameters of the thyroid function (TSH, fT4, ioduria, I/CR) are presented in Table 2.

The TSH level was the only factor that was significantly correlated with the thyroid volume and this correlation was negative (R = −0.28; *p* = 0.0004). In the multivariate linear regression, the correlation of thyroid volume with TSH was independent of the BMI in the population studied. In addition, in the comparison of the thyroid function parameters between the participants, with the thyroid volume in Q1 (<7.9 mL) vs. Q4 (>12.2 mL), the TSH level was the only factor that presented statistical significance (*p* = 0.0132). In women with the thyroid volume in Q1, the median TSH level (minimum–maximum) was higher than in women with the thyroid volume in Q4: 2.09 (0.91–4.56) µIU/mL vs. 1.73 (0.72–4.33) µIU/mL. There was no relationship between the thyroid volume and fT4, ioduria and I/CR. All results are shown in Table 3.

### 3.2. Determination of the Lower Limit of the Thyroid Volume for TSH > 2.5 µIU/mL

**Figure 2:**
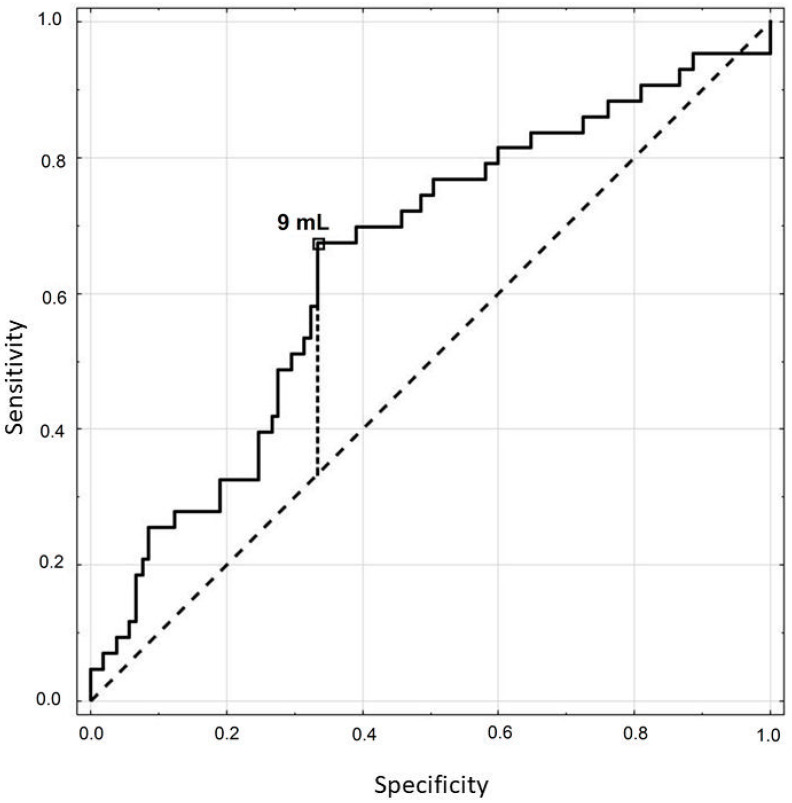
ROC curve for the thyroid volume in estimating if thyrotropin (TSH) is above 2.5 µIU/mL.

The optimal lower limit for the thyroid volume for TSH above 2.5 µIU/mL was determined to be 9 mL (Figure 2). This thyroid volume predicted the level of TSH with the sensitivity of 67%, specificity of 67% and accuracy of 67%. The area under the curve (AUC) was 0.646 (*p* = 0.0037).

## 4. Discussion

There are a variety of studies describing different factors that may affect the volume and function of the thyroid gland. Beyond genetic [19] and environmental factors, such as smoking [10,20], alcohol consumption [20], endocrine disruptors (polychlorinated biphenyls [21], bisphenol A [22]) or cell phone radiation [12]; age and anthropometric parameters are also mentioned [8,9,10,23,24]. It seems that many of these factors interact with each other in a complex way [25]. The most common reason of hypothyroidism is chronic lymphocytic thyroiditis (Hashimoto disease). However, hypothyroidism may also be diagnosed in people without AITD. In some of these patients “small thyroid gland” could be observed. We focused on young women, as subclinical and overt hypothyroidism are especially harmful for women of reproductive age, due to the risk of adverse pregnancy outcomes [1,2,3]. According to the guidelines of the American Thyroid Association and European Thyroid Association, the upper reference limit for TSH in early pregnancy was defined as 2.5 mU/L [4,5]. Young women with TSH > 2.5 mU/L suffer more often from infertility or adverse events in pregnancy [1,2,3]. It is still a matter of doubt, which level of TSH should be the cut-off point in pregnancy, and when levothyroxine substitution should be advised. There are also discrepancies in recommendations for screening for thyroid dysfunction before and during pregnancy from universal screening of TSH, to identify individuals at “high risk” for thyroid illness [4,5,6]. In our opinion, “small thyroid gland” not associated with AITD, is one of the reasons of hypothyroidism.

The first purpose of this study was to analyze the relationship between the thyroid volume and the thyroid function. The second aim was to establish the lower limit for the thyroid volume (“small thyroid gland”), below which, the TSH level is high enough to consider levothyroxine therapy according to the guidelines for pregnant women, or women planning pregnancy. To our knowledge there were no similar studies.

We proved that the thyroid volume was negatively correlated with TSH. In the comparison between participants with the thyroid volume in the 1st quartile vs. the 4th quartile for the study population, the TSH level presented statistical significance. There is little data in the literature on the relationship between TSH and the thyroid volume [10,19,20,26,27,28]. In accordance with our study, the negative correlation between the thyroid size and TSH in women was reported by Barrere et al. [10] in a large study on 2987 French adults, aged 35–60 years. The group of females aged between 35 and 39 consisted of 226 persons and is similar to our study. However, the authors did not test the presence TPOAb and/or TGAb as was done in the present study. Similarly, Hansen et al. [19] and Gomez et al. [20] reported the slight but significant negative correlation between the thyroid size and TSH. However, the study design of Hansen et al. [19] was different from our study as it considered self-reported healthy twins. In the study of Gomez et al. [20] included a smaller group of women (*n* = 134) whose age ranged from 15 to 70 years, with the median of 40 years. On the contrary, Berghout [26] and Feldt-Rasmusen et al. [27] did not observe any relationship between TSH and the thyroid volume in females. However, both studies were conducted in smaller groups of female participants and were carried out in the 1980s, when ultrasonography had a higher risk of error because of the lower accuracy of the ultrasound equipment.

Trimboli et al. [7] in the retrospective study of 434 adult patients, proved that thyroid volume of subjects with normal laboratory results was significantly (*p* < 0.05) higher than thyroid volume of patients with elevated TSH. That is in agreement with our findings. It is worth emphasizing that our study excluded women with positive results of TPOAb and/or TGAb, and hypoechoic/inhomogenous thyroid glands in ultrasound; which made the group very homogenous.

In this study, no relationship between the thyroid volume and fT4, ioduria and I/CR were observed. In the literature there is conflicting information concerning the relationships between the thyroid volume and thyroxin [10,19,26,27] or ioduria and I/CR [10,26].

The question is, if it is worth considering the thyroid volume evaluation in women of reproductive age as an additional screening tool? In our opinion “small thyroid gland” is a relatively frequent problem in women without AITD, and the ultrasonography of the thyroid before pregnancy, seems to be rational during target case finding procedure. Considering that the thyroid volume is negatively correlated with TSH, and even subclinical hypothyroidism may affect fertility and pregnancy outcomes; finding the lower safe limit for the thyroid volume in reproductive women seems to be crucial. The 9 mL cut-off point of the thyroid volume was the predictive factor for TSH level > 2.5 µIU/mL in our study. The prevalence of women with a TSH level above 2.5 µIU/mL and 4.2 µIU/mL (the upper range of TSH) was 29% and 4%, respectively. It means that levothyroxine therapy should be considered in one third of the women from our study in the case of pregnancy.

The limitations of the present study include the relatively small study group. On the other hand, the advantage of the study is a quite homogenous study population, as it consisted of women with no concomitant diseases, at similar age, and living in a similar habitat. Furthermore, the statistical analyses consisted of correlations together with the comparison between two independent groups (women with thyroid volume in the 1st quartile versus in the 4th quartile), making the results more reliable. Further studies with a larger group of women, and with follow-up, would be helpful in a better assessment of the correct meaning of “small thyroid gland”.

## 5. Conclusions

In conclusion, apart from TSH evaluation, ultrasonography seems to be useful in the screening for hypothyroidism in women of reproductive age during target case finding procedure. It is worth considering the use of the term “small thyroid gland” for hypoplastic thyroids if the TSH level is higher than the upper reference limit for pregnancy.

## Figures and Tables

**Table 1:** Characteristics of the study population (*n* = 151).

Characteristics	Mean ± SD or *n* (%)
Age (years)	24 ± 3
Thyroid volume (mL)	10.2 ± 3.2
Presence of thyroid focal lesions	46 (30)
TSH (µIU/mL) (normal range: 0.27–4.2)	2.1 ± 0.9
TSH > 4.2 µIU/mL	6 (4)
TSH > 2.5 µIU/mL	44 (29)
fT4 (pmol/L) (normal range: 12–22)	16.2 ± 2.2
ioduria (µg/L) (normal range: 100–300)	121.3 ± 108.5
I/CR	88.1 ± 111.4
BMI (kg/m^2^)	21.1 ± 2.8
Smoking	
current	16 (11)
former	38 (25)
never	97 (64)

BMI—body mass index; fT4—free thyroxine; I/CR—ioduria per g creatinine; *n*—number; SD—standard deviation; TSH—thyrotropin.

**Table 2:** Correlations between the thyroid volume and the thyroid function.

	R	*p*
TSH (0.27–4.2 µIU/mL)	−0.28	**0.0004**
fT4 (12–22 pmol/L)	0.03	0.6971
ioduria 100–200 (µg/L)	−0.07	0.4196
I/CR	−0.08	0.3314

fT4—free thyroxine; I/CR—ioduria per g creatinine; TSH—thyrotropin. Correlations were performed with Spearman’s rank correlation coefficient for non-parametric data or with Pearson correlation coefficient for parametric data. Results were claimed statistically significant with *p* value < 0.05 (bolded).

**Table 3:** Comparison of the thyroid function parameters in patients with the thyroid volume in the 1st quartile (<7.9 mL) versus the 4th quartile (>12.2 mL).

	Thyroid Volume in Q1 [Median (min–max)]	Thyroid Volume in Q4 [[Median (min–max)]	*P*
TSH (0.27–4.2 µIU/mL)	2.09 (0.91–4.56)	1.73 (0.72–4.33)	**0.0132**
fT4 (12–22 pmol/L)	15.7 (11.4–19.6)	16.2 (11.1–20.7)	0.3859
ioduria 100–200 (µg/L)	109 (30–304)	91 (13–257)	0.6297
I/CR	74 (29–389)	66 (17–163)	0.4679

fT4—free thyroxine; I/CR—ioduria per g creatinine; min—minimum; max—maximum; Q1—the first quartile of the thyroid volume for the entire group (<7.9 mL); Q4—the fourth quartile of the thyroid volume for the entire group (>12.2 mL); TSH—thyrotropin. Comparisons were performed with Mann–Whitney U test for non-parametric data or with a Student t test for parametric data. Results were claimed statistically significant with a *p* value < 0.05 (bolded).

## Data Availability

All datasets used in statistical analyses in this study are available at the request of the reader.
